# Efficacy of calcium hypophosphite toothpaste in remineralizing initial caries in vitro

**DOI:** 10.1186/s12903-026-08959-3

**Published:** 2026-06-18

**Authors:** Bennett Tochukwu Amaechi, Kelly Yang, Amos Chinedu Obiefuna, Temitope Olabisi Omosebi, Pascal Fandrich, Erik Schulze zur Wiesche, Joachim Enax

**Affiliations:** 1 Department of Comprehensive Dentistry, School of Dentistry., University of Texas Health San Antonio, San Antonio, USA; 2 Department of Mathematics and Statistics, University of Maryland Global Campus, San Antonio, USA; 3 Department of Restorative Dentistry, Lagos State University Teaching Hospital, Ikeja, Nigeria; 4 Research Department, Dr. Kurt Wolff GmbH & Co. KG, Bielefeld, Germany

**Keywords:** Calcium hypophosphite, Caries prevention, Remineralization, Toothpaste, Oral care

## Abstract

**Objectives:**

The effectiveness of hydroxyapatite (HAP) toothpastes in remineralizing initial caries and preventing caries development has been well-established. Calcium hypophosphite (CaP) is a calcium compound that readily dissolves in water providing calcium ions, and as such can promote remineralization. The present study investigated the potential of CaP to enhance the caries remineralizing capacity of HAP-containing toothpaste.

**Methods:**

120 bovine tooth blocks were randomly assigned to four toothpaste groups (30/group) containing either 20% HAP, 20% HAP + 1% CaP, 1% CaP, or 1450 ppm fluoride (NaF). Blocks were subjected to 4-day demineralization by plaque growth in a mixed-species microbial caries model for development of initial caries lesions. The lesion-bearing blocks were subjected to remineralization by daily pH cycling consisting of three 2-minute toothpaste treatments, a 2-hour acidic challenge, and then storage in artificial saliva for the rest of the time for 14 days. Toothpastes were homogenized with water (1 toothpaste:3 water) and applied as slurry. Remineralization was measured as a change in mineral density (MD) of the lesions before and after treatment, measured using microcomputed tomography (µCT). Percentage change in MD (percentage remineralization [%Rem]) in each group was calculated. Intra-group (paired t-test) and inter-group (ANOVA/Tukey’s test) comparisons were conducted (α = 0.05).

**Results:**

Paired t-test indicated significant difference (*p*<.001) between pre-treatment and post-treatment MD in all groups demonstrating remineralization. Intergroup comparison based on their %Rem using ANOVA/Tukey’s test showed that 20% HAP + 1% CaP (95.7 ± 3.26) had significantly (*p*<.001) higher %Rem than 1450 ppm fluoride (67.3 ± 7.81), 20% HAP (69.4 ± 4.79), and 1% CaP (80.4 ± 5.49). All other compared groups, except 1450 ppm fluoride vs. 20% HAP, differed significantly (*p*<.001).

**Conclusions:**

The present study shows that the addition of CaP to a HAP-containing toothpaste significantly enhanced the effectiveness of the HAP to remineralize initial caries. It further demonstrates that CaP alone effectively remineralizes caries lesions.

## Introduction

Dental caries represents a major worldwide health issue [1]. Calcium-based strategies aim to raise calcium ion levels in saliva and plaque to tilt the balance toward net remineralization and foster hydroxyapatite formation [2]. Supporting evidence comes from Shaw et al., who found higher calcium and phosphorus levels in the plaque of caries-free children than in that of caries-active children [3]. Examples of frequently used calcium-containing ingredients in oral care formulations include hydroxyapatite (HAP), casein phosphopeptide-amorphous calcium phosphate (CPP-ACP), calcium sodium phosphosilicate (CSPS, or so-called Bioglass), and β-tricalcium phosphate [4].

Within the field of calcium-containing active ingredients, hydroxyapatite, both in microcrystalline and nanocrystalline form, has been clinically tested in various areas of oral care, including remineralization of enamel and dentin as well as reduction of bacterial colonization to enamel surfaces [5]. A recent systematic review with meta-analysis synthesized in vivo and in situ data on hydroxyapatite for caries prevention and found that hydroxyapatite is an efficient alternative for fluoride for all age groups, including young children [6].

Various studies using e.g., microradiography or surface microhardness testing have demonstrated the effectiveness of hydroxyapatite in the remineralization of early caries lesions [7, 8]. It was shown that hydroxyapatite remineralizes subsurface lesions in enamel and dentin [9]. In addition, an in situ study has demonstrated that hydroxyapatite is efficient in remineralizing molar incisor hypomineralization (MIH) [10]. As shown in another in situ study, hydroxyapatite is capable of remineralizing deep enamel defects and forming a more uniform remineralization layer, whereas agents such as fluoride often concentrate effects at the surface layer, limiting deeper repair [11]. Also, due to the growing body of research on the potential side effects of fluoride intake [12, 13], fluoride-free calcium-based ingredients for oral care have gained importance [14].

**Fig. 1:**
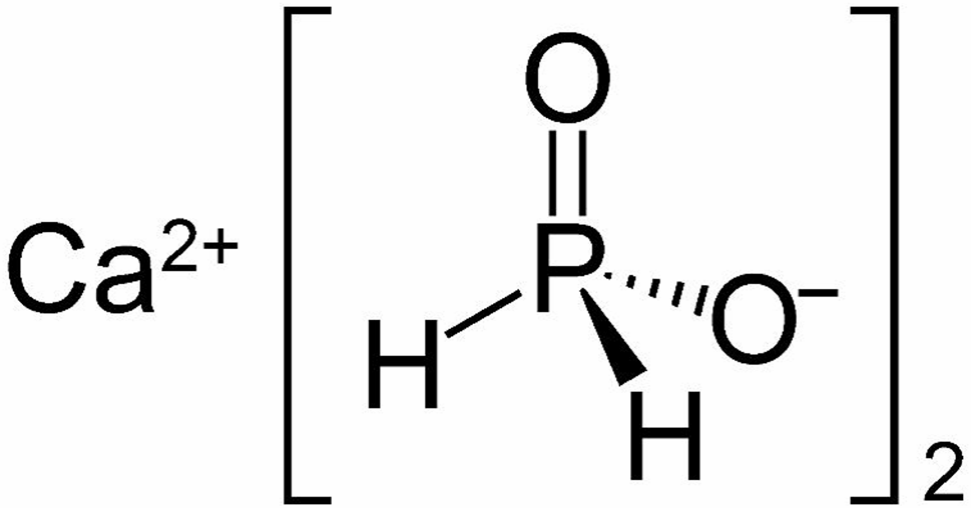
Molecular structure of calcium hypophosphite

A promising calcium agent that has not yet been described for use in oral care formulations is calcium hypophosphite (Ca(H_2_PO_2_)_2_) (Fig. 1). Calcium hypophosphite has a very high-water solubility of 154 g/L at neutral pH [15]. Therefore, in aqueous media it exists as dissolved calcium and hypophosphite ions, with no particulate calcium hypophosphite present.

In contrast to calcium hypophosphite, calcium phosphates such as the active ingredient hydroxyapatite releases calcium ions only under cariogenic (acidic) conditions [16]. This difference in solubility may influence the remineralization efficacy.

While in scientific literature calcium hypophosphite is commonly used as the term for this molecule [15, 17, 18], it is also known as calcium phosphinate [19].

Calcium hypophosphite has been described as a suitable calcium supplement as early as in a publication from 1949 [17]. Besides, it has significant calcium content per molecule (23%), a neutral taste [17], and is safe for humans [20]. It has received a GRAS (Generally Recognized As Safe) status from the FDA U.S. Food & Drug Administration [21]. Finally, calcium hypophosphite is used to treat calcium deficiency in animals [22].

Remineralization performance of toothpastes is frequently investigated in vitro, enabling qualitative imaging and quantitative endpoints within controlled conditions. One of the most relevant techniques for analyzing remineralization effects involves the use of microcomputed X-ray tomography (µCT). Micro-computed tomography provides non-destructive, high-resolution assessment of tooth samples, yielding both qualitative images and quantitative mineral-density metrics [10, 23–25]. This approach enables evaluation of induced caries lesions pre- and post-treatment and supports calculation of percent mineral gain. While there are many different methods for creating artificial caries lesions for in vitro studies such as using acidic solutions and acidic gels [26], microbial caries models have especially the potential to create caries lesions that closely resemble those occurring naturally [27–29].

While calcium phosphates have been studied as remineralizing agents in the dental literature [4], calcium hypophosphite has not been studied in the field of oral care before. Since calcium phosphates exhibit low water solubility at neutral pH, there is a need to evaluate the efficacy of calcium hypophosphite, which is highly water-soluble, and to determine how its solubility affects remineralization efficacy.

The aims of the present in vitro study were (1) to test the potential of a toothpaste containing 1% calcium hypophosphite to remineralize early caries lesions induced by a microbiological caries model compared to toothpastes containing 20% microcrystalline hydroxyapatite or sodium fluoride (1450 ppm fluoride), using µCT, and (2) to test possible synergistic effects of a toothpaste containing 1% calcium hypophosphite and 20% microcrystalline hydroxyapatite, i.e., to test if calcium hypophosphite can enhance the caries remineralization efficacy of hydroxyapatite toothpaste. The first null hypothesis was that calcium hypophosphite would not remineralize initial caries to a percentage greater than zero. The second null hypothesis was that calcium hypophosphite would not have a synergistic effect with hydroxyapatite in terms of remineralization efficacy and that, as a consequence, the initial caries remineralization potential of the two toothpaste formulations would not differ significantly.

## Materials and methods

### Sample preparation

After approval (TR202500000010) by the Institutional Animal Care and Use Committee of the University of Texas Health San Antonio, freshly extracted bovine incisors were procured (Animal Technologies, Tyler, TX, USA; Lot# 8-210519), cleaned with a fine pumice slurry using an electric toothbrush (Braun Oral-B Plaque Remover 3D), and subsequently screened by transillumination [27, 30]. Selected teeth without cracks, hypomineralization, white spot lesions, and other malformations were stored in 0.1% thymol solution until use [27, 30]. Using a water-cooled diamond wire saw (WELL (Walter Ebner Le Locle) Diamond Wire Saws SA, Le Locle, Switzerland), a total of 120 tooth blocks, (approximately 3 mm length x 3 mm width x 1.5 mm thick) were produced from the labial surface of the crown of the teeth. Then all surfaces of each block were coated with two layers of acid-resistant nail varnish (Revlon Consumer Products LLC, New York, NY, USA) except the buccal enamel surface that was submitted to acid challenge for the development of initial caries [26]. 

### Creation of Initial caries lesion

Initial caries lesion was created on the buccal surface of each tooth by subjecting the block to 4-day demineralization using a validated microbial caries model [27–29]. Following revival of the glycerol stock culture of Streptococcus mutans (NCTC 10449, ATCC, Manassas, VA, USA) and Lactobacillus casei (NCIB 8820, ATCC, Manassas, VA, USA) separately, a suspension of mixed culture of these two bacteria was created with Phosphate-buffered saline (PBS) and standardized spectrophotometrically (Spectronic 20; Bausch & Lomb, Rochester, NY, US) to approximately 1 × 10^7^ cells/mL (Optical Density = 0.25 at 520 nm). The growth of the biofilm of this mixed organism culture on tooth samples was performed using the technique outlined by Amaechi et al.^28^ Briefly, each sample was placed in separate wells of a 24-well microtiter plate containing pasteurized human whole saliva and incubated inside an incubator maintained at 5% CO_2_ and at a temperature of 37 °C for 30 min to initiate acquired salivary pellicle formation. The saliva was voluntarily donated by two members of the research team after obtaining a waiver of IRB approval/consent from the Institutional Review Board (IRB) of the University of Texas Health San Antonio. The donors contributed the saliva by chewing on a gum base and spitting intermittently into plastic vials partially buried in ice blocks. Following pellicle formation, each sample was rinsed with PBS and placed in new 24-well microtiter plates containing Todd Hewitt broth (THB) inoculated with the suspension of the mixed Streptococcus mutans and Lactobacilli casei culture (1 × 10^6^ cells/mL). The tooth samples were then incubated inside an incubator maintained at 5% CO_2_ and at a temperature of 37 °C for 24 h to allow for cell adhesion to the samples (adhesion phase). Then, from day 2, the samples were incubated in bacteria-free THB broth inside an incubator maintained at 5% CO_2_ and at 37 °C for 3 additional days. This culture produces a natural cariogenic biofilm, which is subjected to the daily cycle of fasting and feasting as occurs in the oral cavity by cycling it between 10% sucrose solution (6 minutes) for 3 times daily and growth media for the rest of the day. The activity of the Streptococcus mutans and Lactobacillus casei during this demineralization process was monitored once each day by checking for a change in pH immediately before and after the application of 10% sucrose solution. The pH of plaque was also monitored at non-feeding time to check the maintenance of neutrality by CO_2_. After 4 days, few samples were used to confirm production of lesions suitable for remineralization process using MicroCT.

### Measurement of the baseline mineral density of initial caries lesion (MD_b_)

To determine baseline lesion mineral density, tooth blocks were MicroCT scanned in a Bruker SkyScan 1172 (Bruker SkyScan, Aartselaar, Belgium). The samples were attached in batches on a thin rectangular acrylic slide that fit easily into the MicroCT sample tube. A tooth cannot be scanned dry if one wishes to measure mineral density, so the tooth samples were wrapped with tissue paper, and then the tissue paper around the samples was moistened with distilled de-ionized water. Then the samples were stabilized inside the tube using spongy foams to prevent movement during scanning and allow their evaluation over time in the same position, and the tube was sealed with Parafilm (American National Can, Greenwich, CT, USA).

Samples were scanned as previously described [24] at 60 kV, 167 µA tube current, 0.35° rotation steps, 7 frame averaging, and 1090 ms exposure time per step. A 0.5-mm aluminum filter was used during scans [31]. The image pixel size was 6 μm. The images were reconstructed using NRecon (Bruker SkyScan, Aartselaar, Belgium) with a polynomial correction algorithm to reduce beam-hardening effects during reconstructions [31, 32]. Following reconstruction, 150 μm (0.15 mm) diameter volume of interest (VOI), the borders of which extend from along the surface of the enamel down to a depth of 150 μm into the enamel towards the dentin, was manually selected in each sample. Enamel MD was determined from this VOI by a single operator, who also selected the VOI in all samples, to eliminate inter-operator differences. To ensure that measurement before and after remineralization was performed on the same spot, the measurement was performed at a mapped out area (150 μm diameter x 150 μm depth) at the center of each sample.

Hydroxyapatite phantoms (Himed, Bethpage, NY, USA) with the densities of 1.6 ± 0.2 g/cm^3^ and 3.11 ± 0.2 g/cm^3^ were also scanned under the same conditions and settings as the test samples and were used to calibrate mineral density analyses as described by Zou et al. [33]. Briefly, for calibration, the attenuation coefficient values of the two phantom VOIs were firstly measured using Bruker-MicroCT CT-Analyser software (Bruker SkyScan, Aartselaar, Belgium), and then the MD of the two phantoms, 1.6 ± 0.2 g/cm^3^ and 3.11 ± 0.2 g/cm^3^, were calibrated against the attenuation coefficient values of the two phantoms, to produce a straight line relationship between MD and attenuation coefficient [24]. The calibration values were permanently saved in the computer (written to the registry file) so that, even if the computer is restarted, the calibration remains in place. This enabled the same calibration to be used for all the samples before and after treatment.

### Test toothpastes

The compositions of the experimental toothpastes tested in the present study are summarized in Table 1. For the experiments, the same toothpaste base was used for all four toothpastes, differing only in the active ingredients. The toothpastes were provided by the sponsor in a blind format, with identical tubes differentiated only by a random code. Unblinding was performed upon completion of the study.

**Table 1:** Compositions of the four experimental toothpastes tested in the present study

Toothpastes	Ingredients list
Toothpaste with 1% calcium hypophosphite (calcium phosphinate)	Aqua, Hydrated Silica, Glycerin, Sorbitol, Aroma, Sodium Myristoyl Sarcosinate, Tetrapotassium Pyrophosphate, Silica, Calcium Phosphinate, Cellulose gum, Menthol, Zinc PCA, Sodium Methyl Cocoyl Taurate, Sodium Saccharin, Phenoxyethanol, Anethole, Sodium Hydroxide, Benzyl alcohol, Propylparaben, Methylparaben, Beta-Caryophyllene, Terpineol, Limonene, Citric acid, Pinene, Sodium Benzoate.
Toothpaste with 20% microcrystalline hydroxyapatite	Aqua, Hydroxyapatite, Hydrated Silica, Glycerin, Sorbitol, Aroma, Sodium Myristoyl Sarcosinate, Tetrapotassium Pyrophosphate, Silica, Cellulose Gum, Menthol, Zinc PCA, Sodium Methyl Cocoyl Taurate, Sodium Saccharin, Phenoxyethanol, Anethole, Benzyl Alcohol, Propylparaben, Methylparaben, Beta-Caryophyllene, Terpineol, Limonene, Citric Acid, Pinene, Sodium Benzoate.
Toothpaste with 20% microcrystalline hydroxyapatite and 1% calcium hypophosphite (calcium phosphinate)	Aqua, Hydroxyapatite, Hydrated Silica, Glycerin, Sorbitol, Aroma, Sodium Myristoyl Sarcosinate, Tetrapotassium Pyrophosphate, Silica, Calcium Phosphinate, Cellulose Gum, Menthol, Zinc PCA, Sodium Methyl Cocoyl Taurate, Sodium Saccharin, Phenoxyethanol, Anethole, Benzyl Alcohol, Propylparaben, Sodium Hydroxide, Methylparaben, Beta-Caryophyllene, Terpineol, Limonene, Citric Acid, Pinene, Sodium Benzoate.
Toothpaste with sodium fluoride (1450 ppm fluoride)	Aqua, Hydrated Silica, Glycerin, Sorbitol, Aroma, Sodium Myristoyl Sarcosinate, Tetrapotassium Pyrophosphate, Silica, Cellulose Gum, Menthol, Zinc PCA, Sodium Methyl Cocoyl Taurate, Sodium Fluoride, Sodium Saccharin, Phenoxyethanol, Anethole, Benzyl Alcohol, Propylparaben, Sodium Hydroxide, Methylparaben, Beta-Caryophyllene, Terpineol, Limonene, Citric Acid, Pinene, Sodium Benzoate.

### Remineralization treatment procedure

Based on their MD_b_ values, the 120 lesion-bearing blocks were stratified among 4 treatment groups (*N* = 30) as shown in Table 1. The blocks were assigned such that there would be no statistically significant difference between the mean MD_b_ values of the groups. Using dental heavy-duty putty, the 30 blocks in each group were embedded in oblong grooves carved inside a cylindrical acrylic rod that is attached to the cover of a 250-ml treatment tube.

All four groups underwent a standardized pH cycling regimen (alternating demineralization and remineralization) designed to approximate key oral conditions in order to assess each toothpaste’s capacity to remineralize initial lesions. Artificial saliva [MgCl_2_.6H_2_O (0.03 g/L), K_2_HPO_4_ (0.804 g/L), KH_2_PO_4_ (0.326 g/L), KCl (0.625 g/L), Calcium lactate (0.385 g/L), Methyl-4-hydroxybenzoate (2 g/L), pH adjusted to 7.2 using KOH] [8, 30] was used as the simulated oral fluid for storage of the samples, while an acidified buffer composed of 2.2 mmol/L KH_2_PO_4_, 2.2 mmol/L CaCl_2_), and 50 mmol/L acetic acid, with pH raised to 4.5 with KOH [8, 30] was used as the demineralizing solution (DS) and served as the acidic challenging medium [8]. The cyclic treatment regimen for each day (Table 2) was as described by Amaechi et al. [8] and consisted of a 2-hour acidic challenge (AC) [8], three 2-minute toothpaste treatment periods, and then storage in artificial saliva (AS). For treatment, the samples in each group were immersed into 200 mL of the treatment medium (AC, AS, or toothpaste) in a 250 ml treatment tube. All treatments were performed with the treatment medium on a laboratory rocker (Labnet Rocker; Stellar Scientific, Baltimore, MD, USA) at 350 rpm and inside a Reach-in incubator at 37 °C. The pH of each medium was measured once daily before treatment. After treatment with one medium, the specimens were rinsed with running deionized water and dried with a paper towel before immersion into the next medium. The daily regimen was repeated for 14 days.

**Table 2:** pH cycling treatment sequence for the experiment

Daily Events	Treatment
2 min	Toothpaste treatment
4 h	Storage in artificial saliva
2 h (12 Noon)	Acidic challenge
2 min	Toothpaste treatment
4 h	Storage in artificial saliva
2 min	Toothpaste treatment
Till 8:00 A.M. next day	Storage in artificial saliva

### Post-remineralization mineral density of initial caries lesion

After all treatment cycles, post-treatment mineral density (MDt) of each lesion-bearing block was quantified by µCT. This yielded paired baseline (MDb) and post-treatment (MDt) mineral-density values and the corresponding image datasets.

### Efficacy Measurement

µCT data were reviewed to characterize lesion-level remineralization patterns and magnitude per treatment, with pre- and post-images assessed in direct comparison. Within groups, MD_b_ and MD_t_ were compared using paired t-tests to test for significant treatment-related mineral gains. Between-group comparisons used percent change in MD relative to baseline, computed as:

$${\%}\;\mathrm{Change}\;\mathrm{in}\;\mathrm{MD} ({\%}\Delta{\mathrm{MD}})\;=\;[(\mathrm{MD}_{\mathrm{t}}\;-\;\mathrm{MD}_{\mathrm{b}})/\mathrm{MD}_{\mathrm{b}}]\;\times\;100 {\%}$$

### Sample size calculation

The sample size calculations were performed using GPower statistical software and was based on previously published data [7–11]. For the paired sample t-test that compared the mean MD of the lesions before and after remineralization within each group, with a 95% confidence interval and 80% statistical power, it was estimated that an effective sample size of 30 tooth blocks was appropriate for within-group comparison to detect a significant mean difference greater than zero. For the hypothesis that compared the percent remineralization across the four groups, F-test analysis indicated that an effective sample size of 120 was needed to have power greater than 0.80 with a 0.05 significance level to detect a statistically significant difference among the four product groups.

### Statistical analysis

Statistical analyses were conducted in SPSS v28. An alpha level of 0.05 was adopted to define statistical significance for all analyses. Preliminary analyses were conducted to explore the dataset and to check for assumptions violations. Specific t-test assumptions tests including tests for independence, normality and extreme outliers were performed. The assumptions of independence and that of normality were met. The normality assumption was tested using the histogram, Q-Q plot and the Shapiro-Wilk’s test from the tests of normality table and all confirmed that the normality assumption was met for each variable at the alpha level of α = 0.05.

Three analyses were conducted. The first analysis performed before the study procedure was a one-way ANOVA performed to assess if there were significant differences in the mean values of the MD across the four products. The second analysis used one-sided paired t-test to compare the mean MD of the tooth blocks within each group before and after remineralization to determine whether the difference differed significantly from zero. The third analysis used one-way ANOVA to compare the % remineralization across the four product groups. A significant omnibus F-test would lead to a post hoc test to determine specific group means that significantly differed from each other.

## Results

**Fig. 2:**
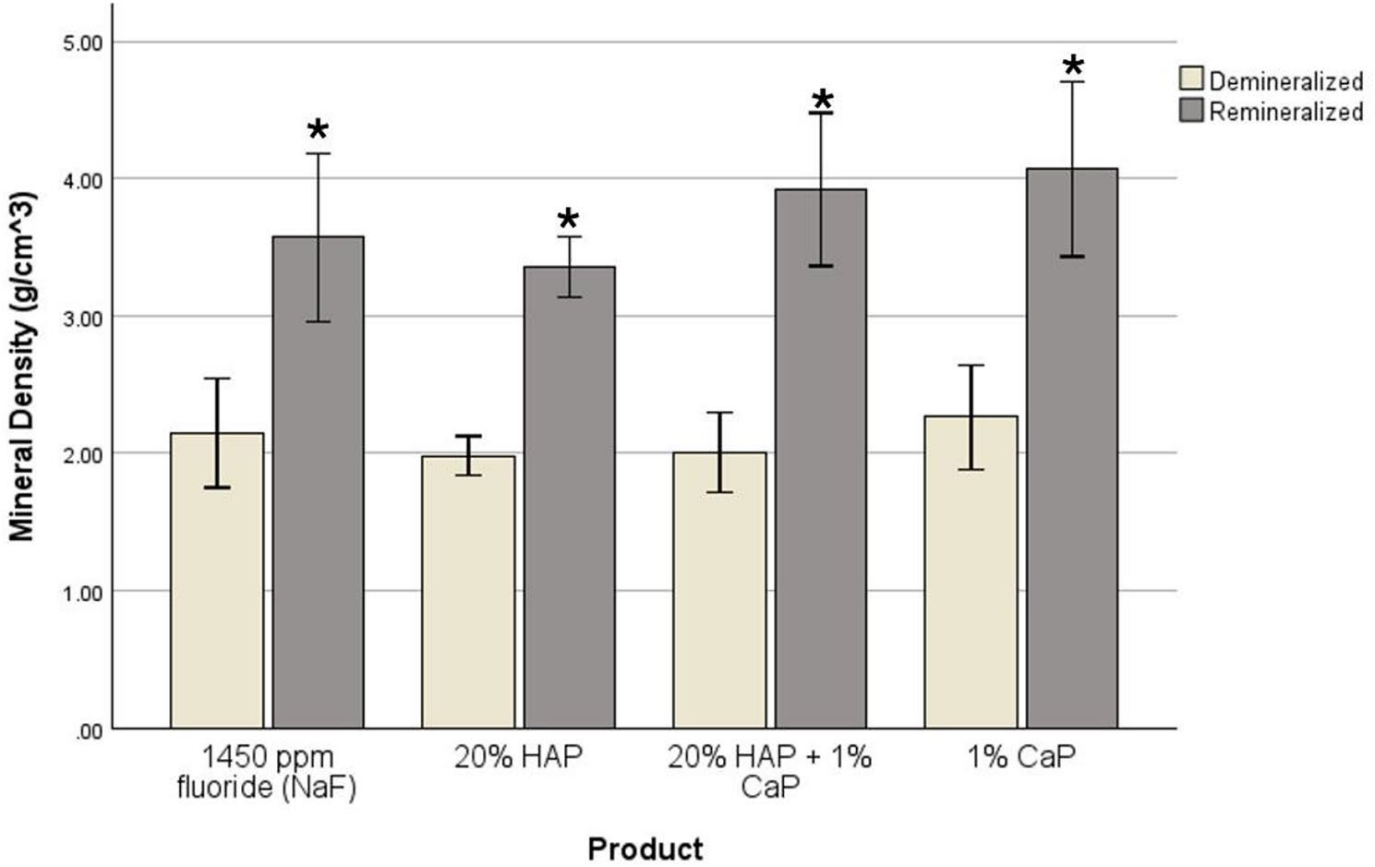
Within group comparison of pre-treatment and post-treatment mean mineral density. *Indicates statistically significant difference between pre-treatment and post-treatment (*p* < .001)

**Fig. 3:**
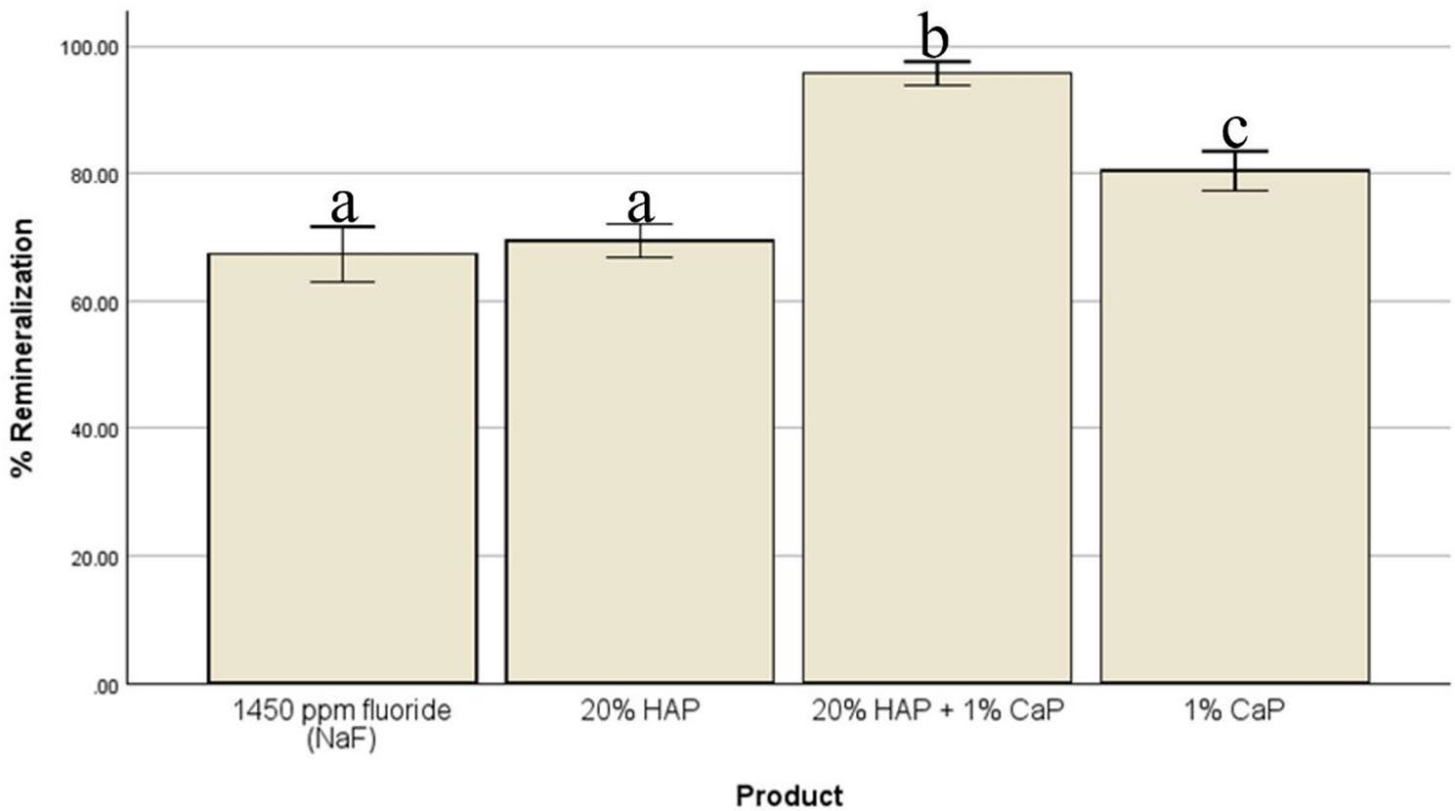
Comparing the treatment groups based on the percentage remineralization achieved with each treatment product. Groups with similar letters (**a**, **b**,**c**) are not statistically significantly different, while different letters indicate statistically significant difference (*p* < .001)

A one-way ANOVA analysis verified there were no statistically significant differences in the mean values of the mineral density among the treatment groups at baseline, F(3,56) = 0.788, *p* = .506. Paired t-test indicated significantly (*p*<.001) higher mineral density in the caries lesions after remineralization than before remineralization in all groups (Fig. 2; Table 3). A one-way ANOVA was applied to compare mean percent remineralization across groups prior to post-hoc pairwise testing. The result of the analysis showed a statistically significant difference in % remineralization among the four groups F(3,56) = 81.30, *p*<.001, at the alpha level of 0.05. Pairwise comparisons of groups using the Tukey test were conducted to identify the specific group means that significantly differed from each other. The result indicated that 20% HAP + 1% CaP (95.7 ± 3.26) had a statistically significantly higher % remineralization than 1450 ppm fluoride (67.3 ± 7.81), 20% HAP (69.42 ± 4.79), and 1% CaP (80.42 ± 5.49) at the alpha level of 0.05 (Fig. 3; Table 4). All other compared groups, except 1450 ppm fluoride (NaF) vs. 20% HAP, differed significantly with respect to the % remineralization (Fig. 3; Table 4).

**Table 3:** Paired Samples T-test comparing the pre-treatment (demineralized) and post-treatment (remineralized) Mineral Density (MD) for each product group. The mean difference is significant at the 0.05 level

Products	Variables (Mean MD±Standard deviation)		Mean Difference	t	df	*P* values	Sig?
	Demineralized	Remineralized					
1450 ppm fluoride (NaF)	2.15 ± 0.71	3.57 ± 1.11	1.42	13.23	14	< 0.001	Yes
20% HAP	1.98 ± 0.25	3.36 ± 0.40	1.37	33.73	14	< 0.001	Yes
20% HAP + 1% CaP	2.00 ± 0.52	3.92 ± 1.01	1.92	15.10	14	< 0.001	Yes
1% CaP	2.26 ± 0.68	4.07 ± 1.15	1.80	14.82	14	< 0.001	Yes

**Table 4:** Tukey HSD Multiple Comparisons of the products based on their achieved mean percentage remineralization (Dependent Variable). *The mean difference is significant at the 0.05 level

(I) Product	(J) Product	Mean Difference ± Std. Error (I-J)	*P* value	95% Confidence Interval	
				Lower Bound	Upper Bound
1450 ppm fluoride (NaF)	20% HAP	-2.11 ± 2.04	0.73	-7.51	3.28
	20% HAP + 1% CaP	-28.39^*^±2.04	< 0.001	-33.79	-22.99
	1% CaP	-13.12^*^±2.04	< 0.001	-18.52	-7.72
20% HAP	1450 ppm fluoride	2.11 ± 2.04	0.73	-3.28	7.51
	20% HAP + 1% CaP	-26.28^*^±2.04	< 0.001	-31.68	-20.88
	1% CaP	-11.01^*^±2.04	< 0.001	-16.40	-5.61
20% HAP + 1% CaP	1450 ppm fluoride	28.39^*^±2.04	< 0.001	22.99	33.79
	20% HAP	26.28^*^±2.04	< 0.001	20.88	31.68
	1% CaP	15.27^*^±2.04	< 0.001	9.87	20.67
1% CaP	1450 ppm fluoride	13.12^*^±2.04	< 0.001	7.72	18.52
	20% HAP	11.01^*^±2.04	< 0.001	5.61	16.40
	20% HAP + 1% CaP	-15.27^*^±2.04	< 0.001	-20.67	-9.87

*. The mean difference is significant at the 0.05 level

**Fig. 4:**
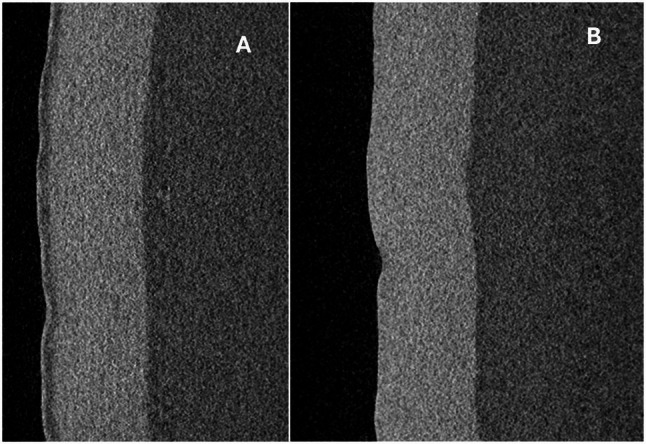
Remineralization of initial caries lesions in bovine tooth blocks with 1450 ppm fluoride (NaF). Representative µCT images of the initial caries lesions, before (**a**) and after (**b**) remineralization by pH cycling. Image pixel size was 6 μm

**Fig. 5:**
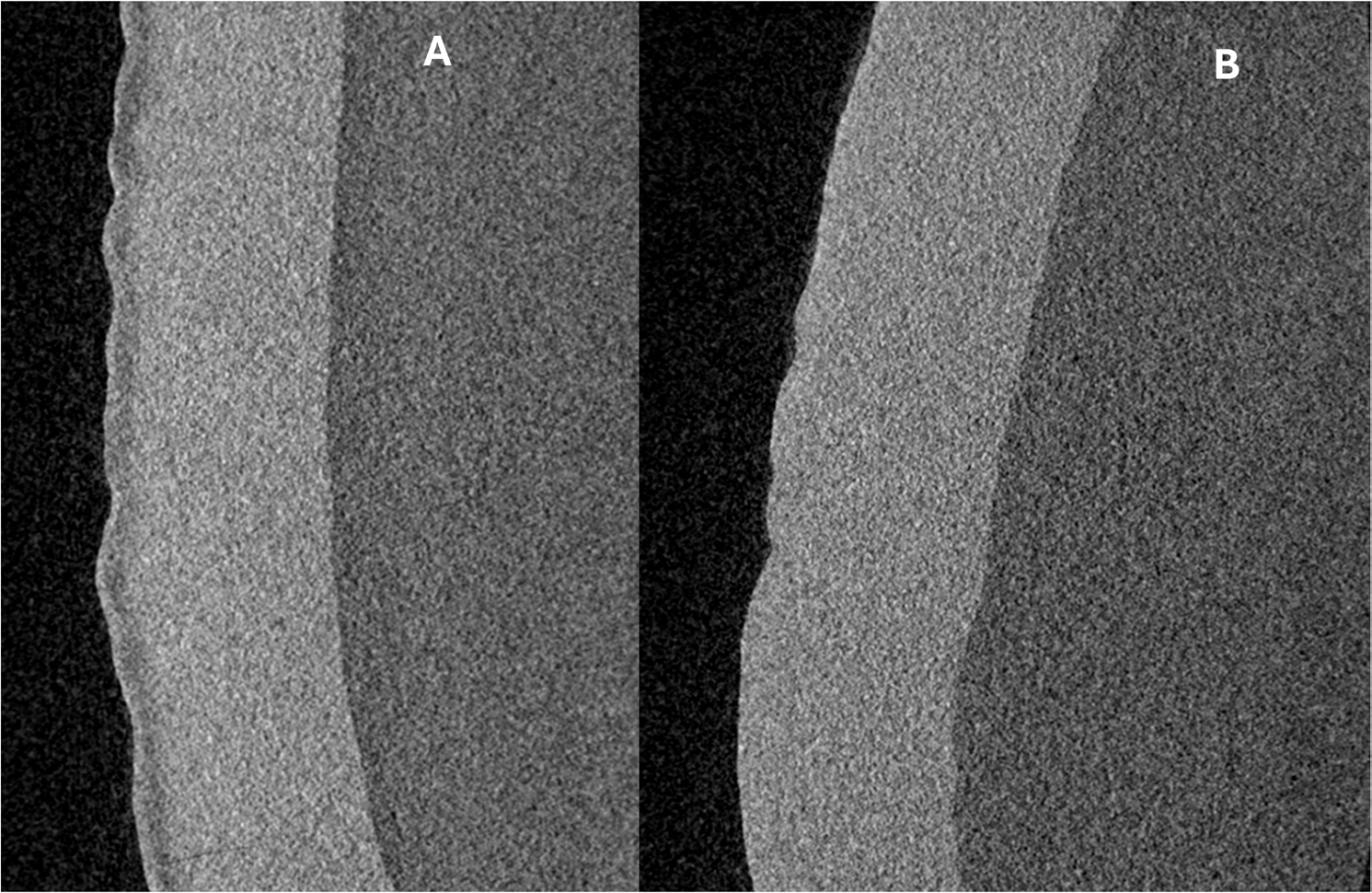
Remineralization of initial caries lesions in bovine tooth blocks with 20% hydroxyapatite toothpaste (HAP). Representative µCT images of the initial caries lesions, before (**a**) and after (**b**) remineralization by pH cycling. Image pixel size was 6 μm

**Fig. 6:**
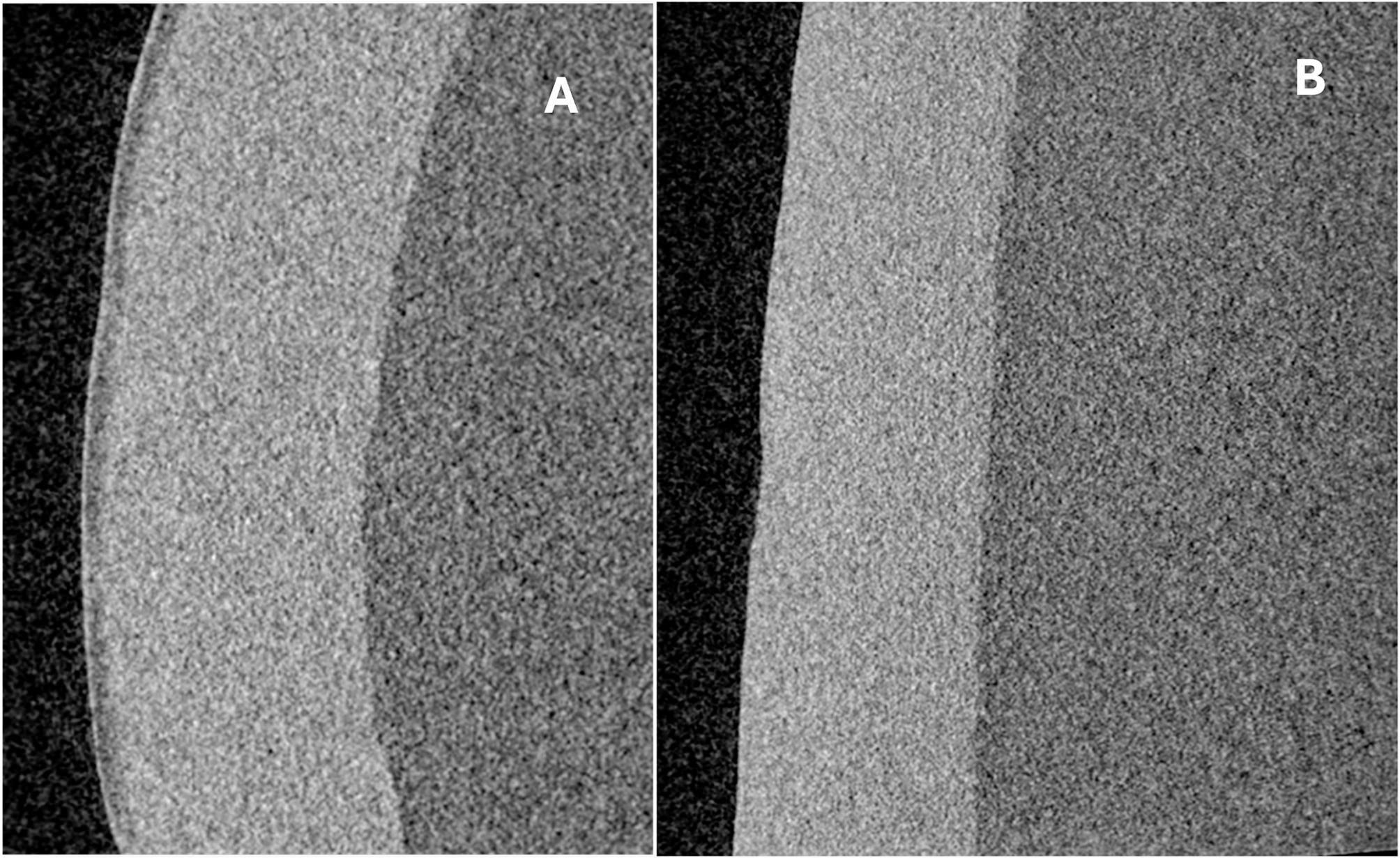
Remineralization of initial caries lesions in bovine tooth blocks with toothpaste containing 20% hydroxyapatite (HAP) plus 1% calcium hypophosphite (CaP). Representative µCT images of the initial caries lesions, before (**a**) and after (**b**) remineralization by pH cycling. Image pixel size was 6 μm

**Fig. 7:**
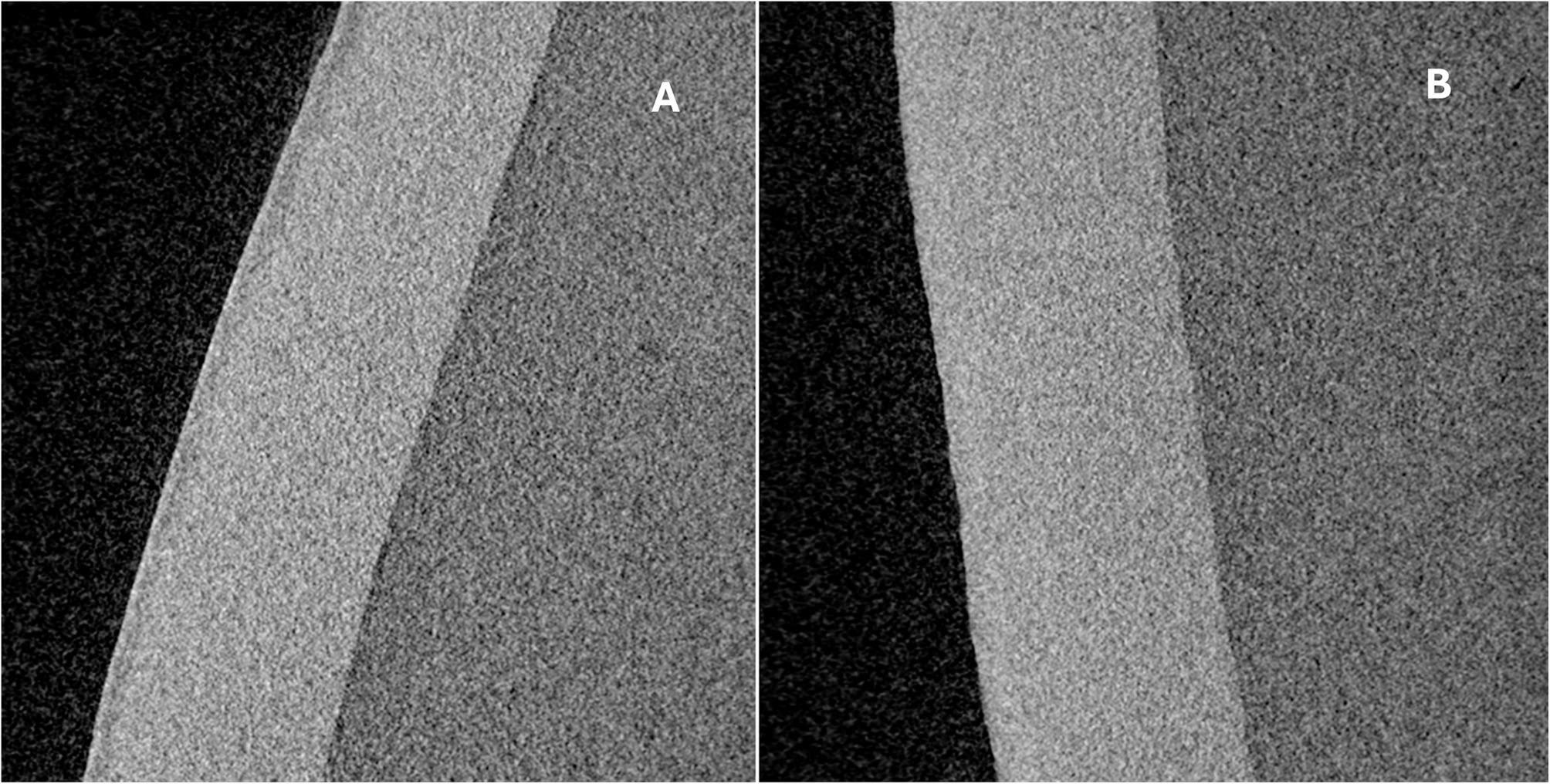
Remineralization of initial caries lesions in bovine tooth blocks with toothpaste containing 1% calcium hypophosphite (CaP). Representative µCT images of the initial caries lesions, before (**a**) and after (**b**) remineralization by pH cycling. Image pixel size was 6 μm

Figures 4, 5, 6 and 7 show the representative microtomographic images of the initial caries lesions before and after remineralization. Due to the limitation of the resolution of the microcomputed tomography (6 μm), visual examination of the images could not effectively depict the variation in percentage remineralization among the product groups (Figs. 4, 5, 6 and 7).

## Discussion

The first null hypothesis was rejected because all treatment groups showed a statistically significant increase in mineral density compared with baseline, indicating that calcium hypophosphite (CaP)-containing formulations effectively remineralized initial caries lesions. The second null hypothesis was rejected because the toothpaste combining hydroxyapatite (HAP) and CaP achieved a significantly higher percentage remineralization than HAP alone, demonstrating a synergistic effect between CaP and HAP.

Although fluoride interventions have been shown to have the most consistent benefit in preventing caries development and remineralizing initial (non-cavitated) caries lesions [34, 35], it is most effective at the surface of the lesion [36], leading to surface-zone remineralization at the expense of the lesion body, making full remineralization difficult to achieve [37]. Consequently, there remains a need for agents that can achieve substantive remineralization of early decay to halt progression toward cavitation. A large body of evidence has established the effectiveness of HAP, the active ingredient in hydroxyapatite toothpastes, in remineralizing initial caries lesions [7–11, 38]. HAP has a low water solubility and neutral pH and, consequently, releases calcium ions only under cariogenic (acidic) conditions [16]. But it is known that a calcium compound, calcium hypophosphite (CaP), readily dissolves in water providing calcium ions [15], and as such can promote remineralization. For this reason, the present study investigated the potential of CaP to remineralize initial caries lesions. It further evaluated if CaP could enhance the caries remineralization efficacy of HAP toothpaste. In this study, the efficacy of CaP was compared with those of HAP and NaF.

In the present study, the initial caries lesions were induced using a well-stablished microbial caries model, which has been validated for production of natural caries lesions by plaque growth with a mixed-species microbial consortium [27, 28]. The sensitivity and effectiveness of this model have been demonstrated in several previous studies [29, 39, 40]. Remineralization efficacy was assessed using a validated pH cycling protocol that alternates demineralization and remineralization phases, approximating diet-driven pH shifts and salivary buffering [41, 42]. The protocol emulates key features of caries development and is widely used to benchmark the efficacy and mineral recovery potential of hydroxyapatite and fluoride [8, 42–44]. Prior work shows that in-vitro pH cycling can generate datasets robust enough to justify subsequent clinical trials [45]. Furthermore, it is pertinent to mention that the period of remineralization varies among studies ranging from 14 [8, 44] and 28 [46] days. However, our standard protocol of 14 days used in the present study has been adequate in differentiating between different concentrations of active ingredients with statistically significant differences in both in situ [10, 11, 47] and in vitro [8, 44] studies. Mineral density has been considered as a standard parameter for the determination of demineralization and remineralization in dental caries, and other diseases that cause dental defects, which provides insight into the dynamic changes associated with the three-dimensional spatial distribution pattern of mineral within dental lesions [24, 33, 48, 49]. Therefore, accurate assessment of mineral density (MD) provides information critical to the understanding of mineralization processes of dental changes during initial caries remineralization studies. Microcomputed tomography (µCT) has the potential to assess the mineral density in teeth three dimensionally and nondestructively, without concerns about the mineral loss caused with dehydration steps [10, 24, 48, 49]. Thus, the quantitative and qualitative outcomes in the present study were measured by µCT, an established method for determining mineral density in hard tissues including enamel, dentin, and bone [23–25]. MicroCT resolves subtle enamel density changes with high microscopic precision [10, 50]. Prior studies have shown that µCT metrics track enamel mineralization status and the performance of remineralizing agents [24, 25, 50]. MicroCT is comparable to Transverse Microradiography (TMR), which has long been the gold standard for measuring mineral loss/gain in demineralization and remineralization studies [51, 52]. However, while both methods can quantify mineral content of tooth tissues as well as show image of the internal structure of the tissue to depict remineralization and demineralization [7, 10, 11, 24, 25], MicroCT is nondestructive, and as such, can be used to monitor remineralization longitudinally, unlike TMR that is destructive and cannot be used to measure remineralization over time. However, both methods can neither distinguish between mineral precipitation and actual remineralization nor give elemental composition within the tissue, like Scanning Electron Microscopy (SEM) [53, 54]. Although some studies have been using SEM to assess remineralization, this method can only show mineral precipitation, and when combined with Energy-Dispersive Spectroscopy can give elemental composition on the surface of the tooth sample [53, 54]. The substrate, bovine enamel, was used in the present study as a substitute for human enamel due to their similar mineral compositions, microstructures, and hardness [55–58], and as such they provide a valid model for assessing remineralization treatments [56]. Bovine teeth are also readily and ethically obtainable, which supports their preferential use in laboratory investigations [57].

The findings of the present study demonstrated CaP efficacy in remineralizing initial caries lesions as a sole active ingredient in toothpaste (Fig. 2; Table 3). This is not unexpected considering that CaP is known to have a very high-water solubility at neutral pH [15], and with its significant calcium content per molecule (23%) [17], it readily provides bioavailable calcium ions even at neutral pH. It has been well-established that bioavailable calcium ions promote the remineralization of demineralized teeth [4, 59–63]. An ideal remineralization active ingredient should deliver calcium ions into the (sub)surface lesion or boost the remineralization properties of saliva and oral reservoirs without any risk of toxicity [64]. The present study demonstrated that CaP met this definition, and thus, the high solubility of CaP in a toothpaste is of great clinical importance, in that when applied intraorally and it comes in contact with saliva, it would not only rapidly release bioavailable calcium ions, but it would also lead to deposits on the tooth coronal enamel, within the root dentinal tubules and available dental plaque, to provide a reservoir of calcium ions to remineralize the tooth surface as and when required [16, 65]. Recent experiments also showed that CaP can spontaneously react with phosphate in artificial saliva to form crystalline hydroxyapatite [66].

Our data further support that HAP alone in toothpaste promotes remineralization of early lesions and limits caries development, consistent with extensive prior evidence [7–11]. However, unlike CaP, hydroxyapatite releases bioavailable calcium ions only under cariogenic (acidic) conditions [16], but just like CaP, the application of HAP-containing toothpaste is known to increase the concentration of calcium ions in saliva and plaque, i.e., shifting the remineralization-demineralization equilibrium towards net remineralization and consequently towards hydroxyapatite formation [2, 16]. Based on these characteristics of CaP and HAP, it was not surprising to observe the highest percentage of remineralization with the toothpaste in which HAP and CaP were combined as the active ingredients in the present study. This highest performance was significantly higher than those of CaP alone, HAP alone, and fluoride alone. It is interesting to observe that the efficacy of CaP alone was significantly higher than that of HAP alone, however, this can be attributed to the immediate calcium ions-releasing effect of CaP in a neutral aqueous solution [15, 17], unlike HAP that requires acidic condition to release bioavailable calcium ions [16]. Thus, the key difference between CaP and calcium phosphates regarding remineralization efficacy lies in the availability of calcium ions: while in HAP the calcium ions are fixed within the apatite lattice, CaP is readily soluble and provides calcium ions in solution.

Likewise, it is not unexpected that CaP achieved significantly greater efficacy in caries remineralization than fluoride alone. It has long been shown in previous studies that fluoride needs calcium to effect remineralization [67–70], which in this study was provided by the artificial saliva with its limited calcium ions compared to CaP.

The observations of the present study are consistent with prior reports showing that HAP toothpastes perform comparably to fluoride formulations for remineralizing initial lesions and limiting lesion progression [7, 11, 42, 71]. Fluoride has the largest amount evidence supporting its caries remineralization efficacy, which has long been established [34–37, 72, 73]. Notably, given the overall high remineralization and the resolution limits of µCT, visual inspection of the images (Figs. 4, 5, 6 and 7) could not discriminate percentage differences between groups; software-based mineral density analysis did so reliably.

While the pH cycling design affords tight control over variables such as pH exposure and treatment frequency, it does not fully capture the complexity of the oral milieu. The absence of salivary proteins, biofilm interactions, and mechanical forces limits the extrapolation of our findings to in vivo scenarios. Another limitation in our study design was the absence of a negative control (placebo), and as such, the effect of pH cycling alone could not be quantified. Furthermore, the depth of lesions was not measured in the present study, since our main objective was improvement in mineral density (remineralization). Nonetheless, our results provide a strong foundation for future clinical studies aimed at validating these outcomes under clinical conditions.

## Conclusions

Calcium hypophosphite is a promising new active ingredient that has not previously been described in the field of oral care. It demonstrates that the addition of calcium hypophosphite to a hydroxyapatite-containing toothpaste significantly enhanced the effectiveness of the hydroxyapatite to remineralize initial caries. It further demonstrates that calcium hypophosphite alone effectively remineralizes caries lesions.

## Data Availability

The datasets generated during and/or analyzed during the current study areavailable from the corresponding author on reasonable request.
